# Vat Photopolymerization 3D Printing in Dentistry: A Comprehensive Review of Actual Popular Technologies

**DOI:** 10.3390/ma17040950

**Published:** 2024-02-19

**Authors:** Elisa Caussin, Christian Moussally, Stéphane Le Goff, Timothy Fasham, Max Troizier-Cheyne, Laurent Tapie, Elisabeth Dursun, Jean-Pierre Attal, Philippe François

**Affiliations:** 1 Faculty of Dental Surgery, University of Paris Cité, 75006 Paris, France; 2 Bretonneau Hospital, Assistance Publique des Hôpitaux de Paris (AP-HP), 75018 Paris, France; 3 Université of Paris Cité, URB2i, 92100 Montrouge, France; 4 Private Practice, 75015 Paris, France; 5 EPF École d’Ingénieurs, 94230 Cachan, France; 6 Henri Mondor Hospital, AP-HP, 94000 Créteil, France; 7 Charles Foix Hospital, AP-HP, 94200 Ivry-Sur-Seine, France

**Keywords:** dentistry, 3D printing, vat photopolymerization, dental additive manufacturing, accuracy

## Abstract

In this comprehensive review, the current state of the art and recent advances in 3D printing in dentistry are explored. This article provides an overview of the fundamental principles of 3D printing with a focus on vat photopolymerization (VP), the most commonly used technological principle in dental practice, which includes SLA, DLP, and LCD (or mSLA) technologies. The advantages, disadvantages, and shortcomings of these technologies are also discussed. This article delves into the key stages of the dental 3D printing process, from computer-aided design (CAD) to postprocessing, emphasizing the importance of postrinsing and postcuring to ensure the biocompatibility of custom-made medical devices. Legal considerations and regulatory obligations related to the production of custom medical devices through 3D printing are also addressed. This article serves as a valuable resource for dental practitioners, researchers, and health care professionals interested in applying this innovative technology in clinical practice.

## 1. Introduction

Three-dimensional (3D) printing, also known as additive manufacturing (AM) in the technology sector, is far from new. Indeed, the principles of this technology were established in the 1980s with the photopolymerization of polymer resin in a vat. While the initial idea and first reported work are attributable to Hideo Kodama, a Japanese researcher, the first two patents on this technology were French and American and were filed within a few days of each other in 1984 [1].

**Figure 1:**
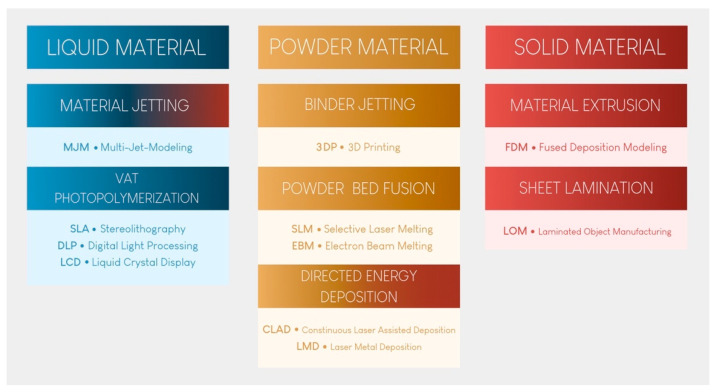
The seven categories of 3D printing technology.

Since then, 3D printing has evolved, and seven major categories of AM have been classified [2]. These seven categories are at the root of many technological procedures currently on the market for processing polymers, metals, and ceramics (Figure 1).

Vat photopolymerization (VP) printing, the oldest technological principle [3], has become the benchmark in dental practice because of its reproducibility, accuracy, cost, and versatility. Material extrusion (MEX) printing, especially fused deposition modeling (FDM), will not be described here, despite its use by some practitioners to produce models. Indeed, the long printing time, high porosity of the materials produced, and absence of stable biocompatible materials make these technologies unsuitable for the long-term production of dental medical devices [4]. Furthermore, it has been shown that this technology produces less accurate materials than the technologies presented and recommended in this article [5,6]. None of the other technologies shown in Figure 1 are currently available in practice.

It is likely that most dentists will possess a 3D printer in the coming years. The affordable acquisition cost of this technology compared to that of subtractive technologies makes 3D printing an ideal complement to intraoral scanners for the on-demand production of certain devices.

The aim of this article is to provide a current and comprehensive review of the application of 3D printing in dental practice. A thorough investigation was conducted by reviewing all the available literature on the subject, focusing on English-language articles accessible through major search engines (PubMed, Embase, and Scopus) and published in prominent indexed journals within the Materials and Dental sector, both with and without impact factors. The results curated in this comprehensive review were extrapolated from this literature search, with reference to the authors’ clinical experience.

In this study, the three technologies currently used for VP printing in dental practice are described, and their advantages, disadvantages, and shortcomings are discussed. The fundamental principles of this family of technologies are explained, and the legislation governing their use is reviewed.

## 2. The Three Technologies for VP Printing

There are three distinct technologies available for achieving VP printing [7]: stereolithography (SLA: stereolithography apparatus), indirect light projection (DLP: digital light processing), and direct light projection using an LCD screen (LCD: liquid crystal display, also called mSLA: mask stereolithography apparatus).

For each printer, unpolymerized, liquid, thermosetting, and photosensitive resin is contained in a vat with a transparent bottom. Other technologies utilize photopolymerization from the top part of the tank. However, since such technologies are not used in the dental field, they will not be described here [4,7]. Different brands rely on various technologies to reduce the adhesion of polymerized pieces, optimize fluid rheology, better withstand the application of heat, and automatically mix the resin in the printing vat, also known as the printing tank. This is a significant field of research and development and explains the high cost of these consumables.

When the printing process begins, the build plate, which is often metallic, is submerged in the vat filled with photosensitive resin. When it approaches the transparent bottom surface, the first layer of resin hardens on the build plate through a photopolymerization reaction [4,7]. When the resin is exposed to specific wavelengths of light, photoinitiators are activated, initiating the polymerization of monomers into polymer chains [8]. The corresponding mechanism is fully comparable to the photopolymerization observed in the application of direct composites in dental procedures.

**Figure 2:**
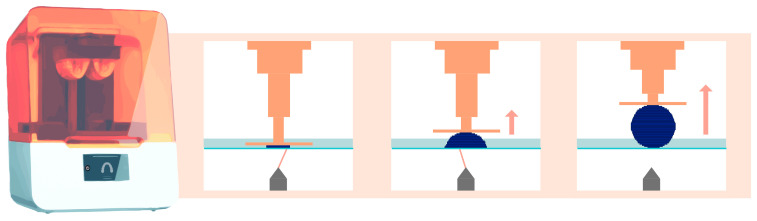
Operation of stereolithography (laser) printing technology. The orange arrow indicates that the platform lifts upward, while the element is being printed.

Then, the build plate lifts vertically along the Z-axis by a few tens of microns. This exact value is defined by the user and corresponds to the layer thickness. The same photopolymerization process then occurs, creating the subsequent layers until the desired object is created [2]. Figure 2 schematically explains this principle for stereolithography technology.

The printer’s resolution can be defined as its ability to reproduce the finest feature of a printed device [2]. This resolution is expressed in the X, Y, and Z axes in µm or DPI (dots per inch), where the Z-axis corresponds to the thickness of each printed layer. The smaller the value is, the more precise the external details of the object are and the longer the printing time becomes [9]. This thickness typically ranges between 25 µm and 200 µm, depending on the clinical indication and desired level of detail. It is impossible to print a layer thinner than the printer’s resolution.

Increasing the vertical printing resolution—for example, from 100 µm to 50 µm—will double the number of layers needed to print the object and double the printing time [4].

### 2.1. SLA Technology

This printing technique relies on the use of a high-energy laser, which draws a cross-section of the object to initiate the photopolymerization reaction of each layer (Figure 2) [2]. This characteristic explains the high isotropy and dimensional stability of the produced elements [10,11].

The set of mirrors inside the laser generator (called the Light Power Unit (LPU)) allows polymerization to occur with consistent resolution at regular intervals on the X and Y axes, regardless of the size and positioning of the object on the build plate. This resolution is directly related to the diameter of the laser generator and the reproducibility of the positioning of the servo-motor systems [7]. This technology enables the simultaneous printing of multiple objects without sacrificing print quality, using a build plate of moderate to significant size.

Objects produced through stereolithographic manufacturing are, therefore, highly accurate [12]. However, for a given printing layer, the focal laser must cross the entire surface; the larger the object to be manufactured or the greater the number of objects to be printed is, the longer the printing time [7].

Form 3B (Formlabs, Somerville, MA, USA), the medical version of Form 3, is the most well-known representative of this category. Form 3BL (Formlabs) enables the use of large quantities of biomedical resin for large-volume printing. The latter is typically used by laboratories.

### 2.2. DLP Technology

**Figure 3:**
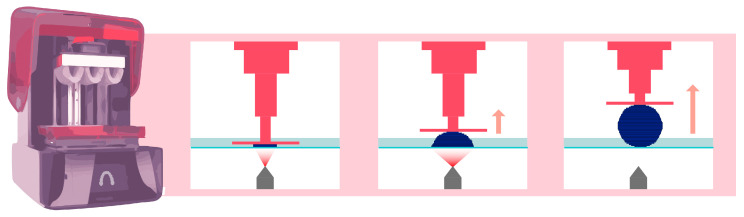
Operation of DLP printing technology. The pink arrow indicates that the platform lifts upward, while the element is being printed.

DLP AM is very similar to SLA technology, as both fall under the category of AM according to the American Society for Testing and Materials (ASTM) [13]. The main difference between the SLA and DLP is the light source (Figure 3). A DLP uses a miniature projector located at a distance from the resin tank; this device is composed of a matrix containing more than a million digital micromirror devices (DMDs), each of which can occupy two positions: one position reflecting light toward the tank and one position reflecting light outside the tank. In this way, a “pixelated” image is projected onto the bottom of the tank [14]. The quantity of mirrors is associated with the resolution of the projected image [15].

As a result, regardless of the printing surface or the number of objects to be printed, the printing time for any given layer thickness of resin is the same [4]. In a dental office, this technology is the only option capable of enabling single-session restorations, which are referred to as chairside restorations.

The majority of DLP chips used in the dental field have a resolution of 1080 p. The larger the projected image is, the lower the resolution will be. In other words, a larger printing surface corresponds to a greater pixel width, which results in a greater approximation of the layer to be printed [14]. This approach remains somewhat theoretical because numerous algorithms and compensation software programs aim to reduce this effect. In dentistry, this approach has little functional impact, as most indications require an accuracy of approximately 100 µm [16]. Nevertheless, this approach may result in a less smooth surface finish than SLA technology [17]. Additionally, for technological reasons, the possible printing volume is generally more limited than that when using SLA technologies [14].

The most common printers include the Sprintray Pro 55S and 95S (Sprintray, Los Angeles, CA, USA), the NextDent 5100 (3D System, Rock Hill, SC, USA), the Varseo XS (Bego, Bremen, Germany), and the CaraPrint 4.0 Pro (Kulzer, Hanau, Germany).

### 2.3. LCD (Also Known as mSLA)

**Figure 4:**
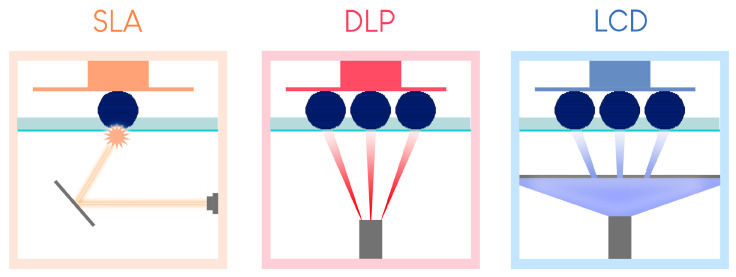
Summary of the differences between the three resin tank polymerization printing processes (version of [23]).

This technology is sometimes associated with and classified as a DLP technology due to the similarities between the two. However, mSLA possesses a major difference. Instead of DLP chips, an LED projector is hidden behind an LCD screen placed near the printing tank (Figure 4) [18]. The projector emits monochromatic ultraviolet light, which is filtered by the LCD screen on the areas not to be printed in the tank [19].

This approach enables higher printing resolutions, with theoretical LCD screen resolutions ranging from 4 to 12 K. These values are, in fact, slightly lower due to an optical convergence phenomenon between two adjacent pixels [20].

However, these printers have limitations caused mainly by overheating. The high light intensities required for layer-by-layer printing lead to significant heating of the LCD screen, and the cooling provided by the fans inside the printer is insufficient to resolve this issue [21]. Moreover, an LCD screen degrades much faster than an SLA or a DLP chip and must be regarded as a consumable to be replaced after a certain number of hours of use. Thus, the print quality gradually declines as the printer is used until a new LCD screen is installed [21]. The light intensity in LCD AM is relatively low, as only 10% of the light can pass through the LCD screen, with the remaining 90% absorbed by the screen. Additionally, as noted earlier, partial light leakage may lead to uneven exposure of the photosensitive resin at the bottom, requiring regular cleaning of the liquid tank [18].

The most common dental-specific printers include the Ackuretta SOL (Ackuretta, Taipei City, Taiwan), the NextDent LCD1 (NextDent, Soesterberg, The Netherlands), and the Sonic 4K 2022 (Phrozen, Taipei City, Taiwan). There are also printers not specific to the dental field, such as the Sonic Mini 8K (Phrozen, Taipei City, Taiwan), Sonic Mighty 12K (Phrozen Taipei City, Taiwan), Creality Halot (Creality, Shenzhen, China), Elegoo Mars (Elegoo, Shenzhen, China), Elegoo Saturn (Elegoo, Shenzhen, China), and Anycubic Photon Mono (Anycubic, Kowloon, Hong Kong).

The recent popularity of such printers is primarily based on their price, which, depending on the brand, is 2 to 10 times lower than that of an SLA or DLP printer, which both have comparable prices. This price difference is explained in part by lower manufacturing costs [22] and also by the lower optimization of nondental-specific LCD printers. Indeed, the latter printers are generally less ergonomic and have few or no certified printing profiles.

### 2.4. Advantages and Disadvantages of Each Technology in the Dental Office

#### 2.4.1. Printing Time

**Figure 5:**
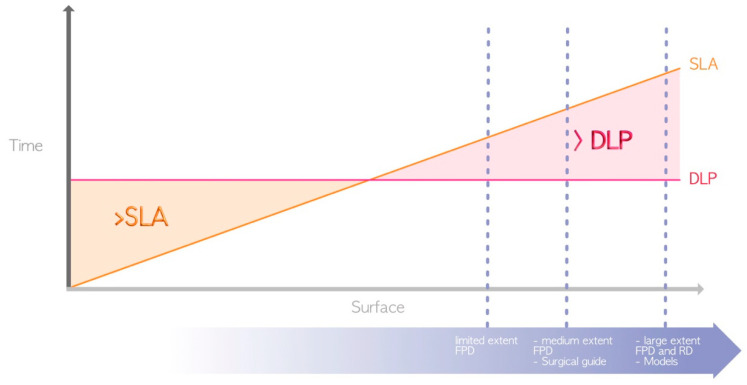
Difference in printing speed between SLA and DLP (due to their great heterogeneity, LCD printers are not represented here). The surfaces required for the manufacture of the various elements represented by the dashed blue lines are located in the area where DLP prints faster. FPD = fixed partial denture; RD = removable denture.

In terms of advantages, DLP and LCD technologies are generally favored over SLA (Figure 5) for the following aforementioned reason: the use of a laser for SLA compared to light projection for LCD/DLP [4]. Thus, DLP and LCD are the only technologies that currently enable the chairside fabrication of restorations, such as fixed prosthetic elements or occlusal splints, in a single session.

Other factors can also impact the printing time. Indeed, printing time depends on the physicochemical and rheological properties of the printed resins. Flexible or semirigid resins have systematically longer printing times than rigid resins, regardless of the technology used. This phenomenon can be explained by the slower movements of the printer used to limit potential deformation induced by the detachment of the printed object from the bottom of the tank after the formation of each printed layer [24]. The size of the build plate and the print vat also play major roles. For a given technology, reducing the size of the build plate and vat can limit the printing time, as the resin can reposition itself more quickly and evenly at the bottom of the tank between the printing of each layer.

**Figure 6:**
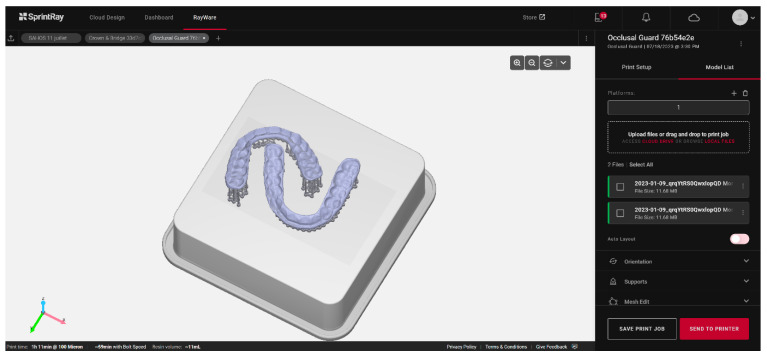
Example of occlusal splint printing planning based on artificial intelligence using the Rayware Cloud software (Sprintray, Los Angeles, CA, USA). After the stl design file was uploaded, and the printing resin was selected (Keysplint Soft, Keystone Industries, Gibbstown, NJ, USA), the positioning of the elements to be printed and the orientation of the splints were automatically generated using artificial intelligence.

The automation of the process, which precludes human intervention on the printer outside regular working hours, can also serve as a variable for optimization. This is the strategy chosen by Formlabs with the Form Auto, which includes the addition of a robot to the printer to detach the printed elements and start a new print. The productivity gain obtained with this method can compensate for slower unit printing, unlike in DLP or LCD (Figure 6).

#### 2.4.2. Printing Accuracy

**Figure 7:**
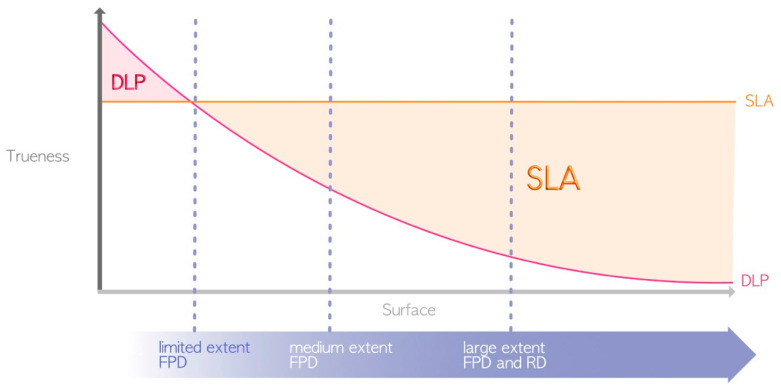
Differences in printing accuracy between SLA and DLP (due to their great heterogeneity, LCD printers are not represented here). FPD = fixed partial denture; RD = removable denture.

The term “accuracy” encompasses the concepts of precision and trueness. The precision or repeatability of a 3D printer denotes its ability to produce objects with consistent dimensions, i.e., how closely repeated prints match each other. Trueness, on the other hand, pertains to the difference between the printed object and the actual dimensions of the desired object [25]. Due to variations in protocols, selected technologies, printer parameters, and the 3D polymer materials utilized, comparing results across different studies is challenging. As mentioned earlier, SLA allows one to print with constant trueness regardless of the printing volume. DLP has variable precision depending on the printing volume, although this precision is partially compensated for by an algorithm, while LCD suffers from optical convergence. In theory, for minimal printing volumes, DLP seems to present greater trueness than SLA [26,27,28] because the high-resolution projection of DLP on a small surface has a greater resolution than that of the SLA laser. However, in general, the SLA can be considered more precise than the DLP or LCD [12,29,30,31] (Figure 7), although this precision affects only the surface finish and not the quality of adaptation or insertion. LCD printing technology seems inferior to DLP printing [18,32], but too few studies have evaluated the accuracy of LCD 3D printing technology [22].

#### 2.4.3. Ease of Use

The ease of use is not correlated with the employed technology but instead with the ergonomics of the software and devices used. Because each brand has its advantages and disadvantages, generalizations should be cautioned [33]. Low-cost LCD printers are often less ergonomic than high-cost printers and use open-source software and unautomated printing and postprocessing devices. Additionally, specific printing parameters for each resin may not always be readily available and need to be determined by the practitioner.

Overall, systems specifically developed for the dental field aim to simplify the use of these materials. Examples include the use of cartridge and automixing tray systems to avoid direct resin handling, artificial intelligence software to launch the printing process quasi-automatically (Figure 7), and preprogrammed postprocessing devices for various resins. These elements are crucial for smooth and reproducible daily use.

## 3. Fundamental Principles of Vat Polymerization 3D Printing

### 3.1. The Slicer

A 3D file must be sliced to be printed layer by layer. This process is achieved through software known as a “slicer”, which transforms a 3D file (most commonly in the stl format) into a sequence of 2D files [34]. Each manufacturer typically has its own proprietary slicer software.

**Figure 8:**
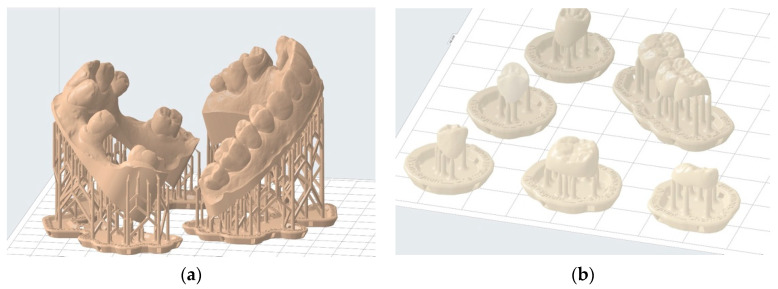
Examples of printing support structure placement: (**a**) correct positioning of printing supports to print two hollowed full wax-up models—the entire useful upper surface is free of support structures; (**b**) incorrect positioning of printing supports for temporary shells—prosthetic element contact with the intrados should be minimized.

This software enables the selection of printing parameters such as layer thickness and resin type. Mainly, this approach allows for the addition of print support structures that increase contact with the build plate [34]. This process prevents the printed object from detaching during vertical movements. Support structures also help minimize deformations during printing through their strategic placement on areas that leave the functionality of the printed object unaffected. In some cases, it is possible to completely avoid these critical areas [35,36,37]. This approach is applicable, for instance, in study models where occlusal surfaces of teeth and surrounding soft tissues can be entirely avoided (Figure 8a). In other cases, such as occlusal splints, a compromise must be made regarding the placement of such structures on the intrados and extrados surface. Generally, it is advisable to avoid prosthetic intrados as much as possible to avoid interfering with their insertion (Figure 8b).

The first 10–15 layers of the print are intentionally overexposed for a longer period of time than the subsequent layers to ensure good adhesion to the build plate and minimize the risk of failure due to detachment [38]. These layers consequently have relative dimensional inaccuracy, which is negligible when using support structures because the object is positioned at a distance from these initial layers.

**Figure 9:**
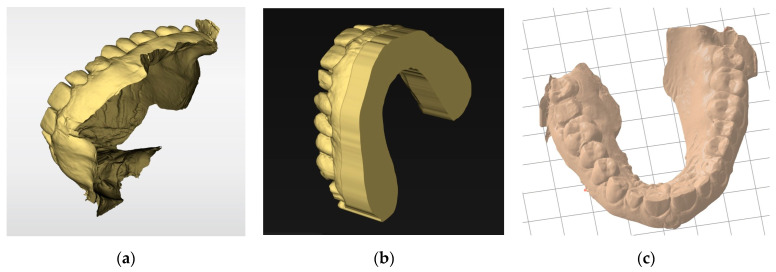
Basing models: (**a**) stl file from an intraoral scanner after acquisition. This non-closed geometry cannot be printed; (**b**) the same impression after cleaning and basing with specific software (Inlab 22, Dentsply Sirona, Charlotte, NC, USA). Numerous parameters are available for adjusting the model geometry or performing die placements. This continuous geometry can be printed; (**c**) the same impression automatically based with a slicer (PreForm, Formlabs, Somerville, MA, USA). The basing is less efficient but saves time.

The slicer then generates a manufacturing path saved as a G-code file containing all the information needed for the digital control of the printer for each layer. This file is transmitted to and interpreted by the printer. The STL file must have a closed geometry and not be an “open” mesh that would lead to printing failure (Figure 9). In the case of prosthetic designs, the file is inherently closed when exported from the design software. However, optical impressions from an intraoral scanner are often “open” (Figure 9a). Additional software may be required to close these regions (Figure 9b). Some slicers also offer an option to “close” the STL mesh from an optical impression to make it immediately printable. However, while this step simplifies the procedure, such slicers are often still less efficient than dedicated software (Figure 9c).

### 3.2. Build Orientation

The way an object is positioned in the slicer has an impact on the printing time, trueness, and number of elements that can be simultaneously placed on the build plate [39]. Similarly, object positioning affects the risk of the object detaching from the build plate during printing, called “warping”. Positioning also influences other properties such as strength, surface morphology, and bacterial response [40,41]. These general principles can vary from one printer to another and from one resin to another.

**Figure 10:**
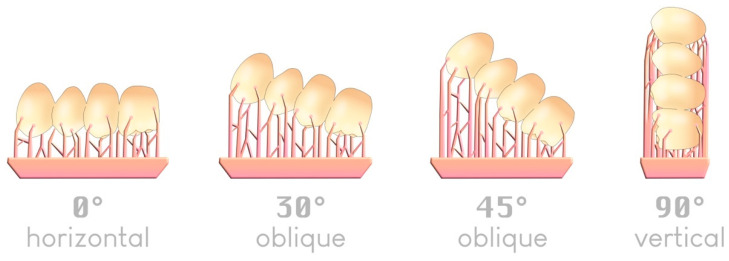
The four typical angles for positioning an item on a build plate; an example of a 4-unit bridge.

There are four typical angles—although an infinite number is possible—by which an object can be arranged in a slicer relative to the build plate (Figure 10):

Regarding printing trueness, the 45° angle relative to the build plate appears in most studies to be the most accurate when printing supports are used [42,43,44,45,46]. A 0° angle was previously reported to offer comparable trueness [47,48].

Regarding printing speed, a 0° angle is the fastest since it requires fewer layers to produce the object. The more vertically inclined the object is (toward 90°), the longer the printing time becomes [47,49].

Regarding the number of elements to print, the 90° angle allows one to place a larger number of printable elements on the same build plate, which can be attractive for large production volumes [47].

**Figure 11:**
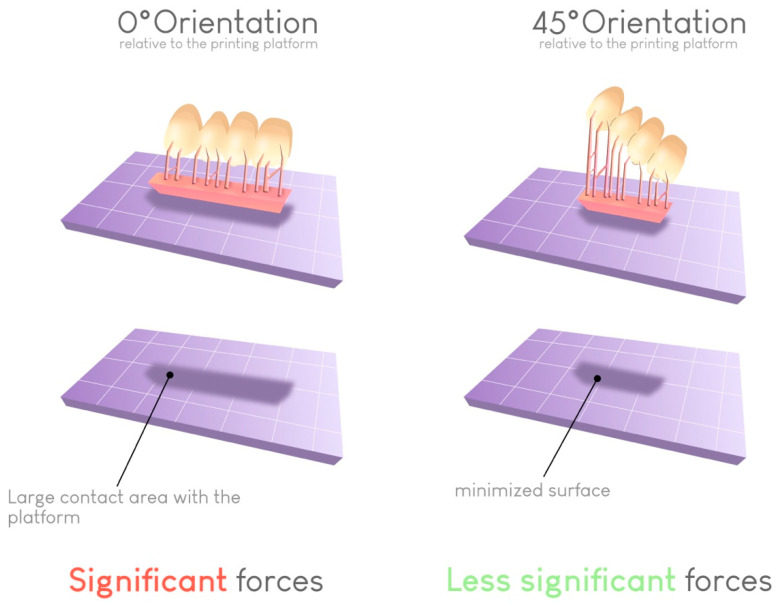
Impact of the build orientation on the reduction in the contact surface for each layer. This reduction reduces the peeling force required to detach the print from the vat at each layer and thus limits the risk of printing failures.

Regarding the risk of warping, a 45° angle results in the lowest failure rate. This angle helps limit the contact surface between the printed object and the build plate at each layer if the support structures are correctly placed. In this way, the forces required for detaching the object from the bottom of the build plate at each transition increase (Figure 11).

Furthermore, the orientation seems to have a relative impact on the mechanical properties of the printed element. However, this effect appears to be more resin dependent, and generalizations cannot be established [50]. The orientation also affects the proper flow of unpolymerized resin. Since the movement of the build plate is only vertical, certain printed areas can behave like reservoirs, leading to an accumulation of unpolymerized resin [51,52]. A judicious choice of orientation for the printed piece can reduce or eliminate this detrimental effect. Many programs take this parameter into account and indicate the areas where unpolymerized resin could accumulate and adversely affect print quality.

**Figure 12:**
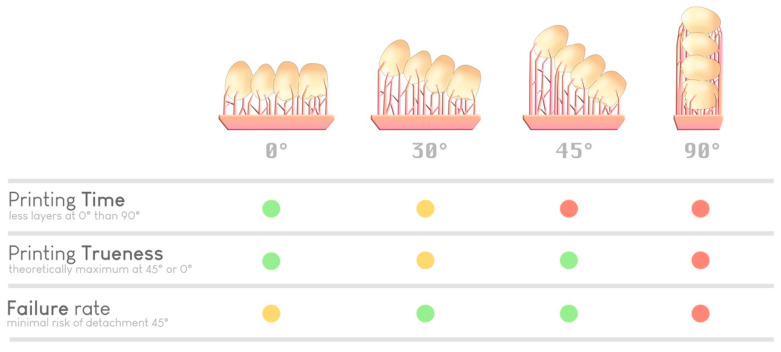
Selection of an optimal orientation based on various parameters (time, precision, and risk of failure). A green dot indicates a good performance regarding a specific parameter, a yellow dot indicates average performance, and red dot indicates inferior performance. There is no ideal orientation; it is the experience of the practitioner (or the artificial intelligence of the slicer) that guides the choice.

Ultimately, with knowledge and experience, the practitioner or prosthetist decides his or her ideal parameters for each print, relying on accuracy or productivity requirements (Figure 12). Notably, some slicers offer a simplified “optimized” angulation based on the number of elements to print, without necessarily considering other parameters.

### 3.3. Printing Materials

The main advantage of AM in the dental field is to produce customized devices in less time at a lower cost [53] thanks to reduced material costs, especially those of resin.

Printing resins are often incorrectly claimed to be similar to polymethyl methacrylate (PMMA) resins when they have compositions similar to those of direct or machinable composite resins.

Resins present several different characteristics. The first is their photoinitiators [54], which are activated by UV wavelengths ranging from 365 to 405 nm depending on the technology and printer. A resin from one brand may not be printable with a printer from another brand if the wavelengths are not compatible. Indeed, each 3D printer emits specific monochromatic light with a characteristic wavelength.

The second characteristic is the filler content, which affects the final mechanical properties of the printed element and the rheological properties of the printing resin [55]. Excessively high filler content can lead to a more significant diffusion of incident light energy [56]. Thus, a certain level of filler content cannot be exceeded if the resin is to remain printable; this is currently one of the limitations of resin vat 3D printing. Indeed, 3D-printed resins represent between 3% and 50% of filler content. The weight percentage of filler by itself does not impact flexural strength. However, the overall composition of the resin can influence this measure. Despite having a lower flexural strength, modulus, and hardness compared to milled and traditional composite and ceramic materials, 3D-printed resins exhibit non-brittle, plastic behavior [57].

Finally, the intrinsic properties of these materials are related to their indications. Each resin has an indication and must be used within that framework. Using a resin for other applications is not legally compliant. Resins intended for the manufacture of surgical guides, for example, must necessarily be biocompatible but also autoclavable to ensure the sterility of the intervention. Thus, such resins must be able to withstand the extreme conditions of these processes and not cause deleterious effects to biological tissues during intervention.

Although printing resins contain well-known monomers such as Bis-GMA, TEGDMA, and UDMA, they also contain less-known monomers directly derived from the chemical industry. This observation, combined with the high opacity of safety data sheets provided by manufacturers and the novelty of this technology, suggests the need for caution in long-term use [58,59], especially because the printed elements are voluminous [39]. The long-term allergic effects on practitioners and patients also remain understudied [60]. Lee et al. [61] found that the double-bond conversion increased significantly when the specimens were printed at a high temperature (70 °C). Moreover, the mechanical properties increased, and the residual monomer levels reduced. These phenomena should be explored in the future.

Printing resins are currently offered by manufacturers for a wide range of dental applications such as study models, surgical guides, orthodontics aligners, occlusal splints, custom trays, temporary and definitive crowns, bonded partial restorations, and temporary and definitive removable dentures. Independent experimental and clinical studies to certify the proposed applications are still lacking [62]. Previous authors particularly emphasized that caution should be taken regarding the use of resins for definitive prosthetic elements that are intended to remain in the mouth for years.

### 3.4. Postprocessing

The printed element cannot be used immediately after printing and requires postprocessing steps. The mechanical, optical, and biological properties of this element are unsatisfactory because the resin is not fully polymerized [63]. Postprocessing is, therefore, crucial, especially for elements intended for intraoral use [64]. Postprocessing consists of five steps [4]:

- Rinsing with a resin solvent to remove unpolymerized resin;
- Drying;
- Postpolymerization in a light device to enhance the properties of the printed object;
- Removal of support structures;
- Optional polishing.

This postprocessing procedure should be of the highest quality. This procedure should also be performed as quickly as possible to integrate it into routine dental care and produce high-quality custom medical devices. Currently, postprocessing is likely the most limiting step in resin vat photopolymerization 3D printing.

#### 3.4.1. Postrinsing

To eliminate any unpolymerized resin around the printed element, the piece is generally rinsed with 99% isopropyl alcohol (IPA) [65]. This solution is very effective but poses a problem in dental offices due to its evaporation in the air and highly flammable nature. Thus, rinsing should preferably be performed under a fume hood or in a well-ventilated space, away from any flammable elements [66,67]. It was shown that prolonged exposure to IPA can irritate the mucosa or cause dermatitis [68]. Other solvents, such as water, tripropylene glycol monomethyl ether (TPM), and other industrial solvents, are available. However, their use has not yet been certified for biomedical applications [69], and these solvents might also have other adverse effects on the properties of the printed elements.

These solvents are placed in an active cleaning device called a wash unit, which aims to agitate the solvent to maximize its effectiveness.

The main purpose of postrinsing is to achieve the desired geometry of the object. In the case of improper rinsing, unpolymerized resin remains on the object and fuses with it during the postpolymerization step. This process can, for example, impact the insertion of an object into the mouth [65]. In addition, too many closely spaced support structures can be detrimental to thorough rinsing.

An additional purpose of postrinsing is to improve the biocompatibility of the printed material. Proper adherence to this step and the indicated times is essential to ensure the safety of a custom medical device. Depending on the brand and type of resin, the rinsing time can vary between 5 and 20 min. This process always entails a compromise between the time needed to achieve the desired solvent effect and the desire not to denature the prepolymerized printed object. An immersion time that is too long alters the mechanical properties of the object due to the absorption of alcohol into the resin matrix of the prepolymerized object, leading to the dissolution of linear polymer chains and, therefore, a decrease in flexural strength [70]. We recommend using at least one separate alcohol bath for biocompatible and non-biocompatible resins to avoid resin mixing.

Rinsing solvents pose an environmental problem, as they become saturated with resin over time and need to be disposed of and replaced with new solvents. The disposal of 99% IPA in wastewater is harmful to the environment [71] and legally prohibited, although this practice remains widespread. In response, two solutions exist and can be used concurrently:

- Decanting of saturated isopropyl alcohol: Due to gravity, sedimentation of unpolymerized resin occurs in the container. Removing the saturated portion allows for the recovery of unsaturated alcohol [72].
- Elimination of saturated isopropyl alcohol through a recycling circuit: This option is offered by waste disposal facilities to individuals. Given the small volume of solvent used, dentists can be considered individuals and thus benefit from this solution. However, it would be useful for manufacturers and distributors to work on organizing the collection of such wastes and recycling solvents that already exist on an industrial scale to limit our environmental impact.

#### 3.4.2. Drying Printed Elements

Once the object is cleaned, it must be actively dried using an air syringe and left for a few minutes to evaporate, eliminating any traces of IPA in the printed object that might be trapped during postpolymerization [73]. The various postpolymerization devices also include a hot air-drying system (Formcure, Formlabs, Somerville, MA, USA) that promotes the evaporation of isopropyl alcohol residues.

#### 3.4.3. Postcuring

Postpolymerization is the second crucial step in postprocessing and allows the creation of printed objects with optimized biological, optical, and mechanical properties [4].

This step often involves a device that provides high light irradiance and heat input. Depending on the brand and type of device, the light irradiance and wavelength vary significantly [74]. Each resin must be used with its recommended device, as wavelength incompatibilities hinder complete polymerization. All the manufacturer’s recommendations must be followed to avoid side effects, especially colorimetric changes due to prolonged treatment [75,76]. A polymerization treatment that is too short or inappropriate can also lead to shade errors in prosthetic elements. A few years ago, postpolymerization took a great deal of time, but this step has now been reduced to a few minutes by most manufacturers that offer equipment for dental surgery, for obvious ergonomic reasons.

After polymerization, the surface layer has poorer mechanical properties due to the inhibition of radical polymerization by oxygen [77]. To overcome this problem, several manufacturers propose performing postpolymerization under a vacuum or nitrogen, which was previously shown to be effective [78].

#### 3.4.4. Support Structure Removal

**Figure 13:**
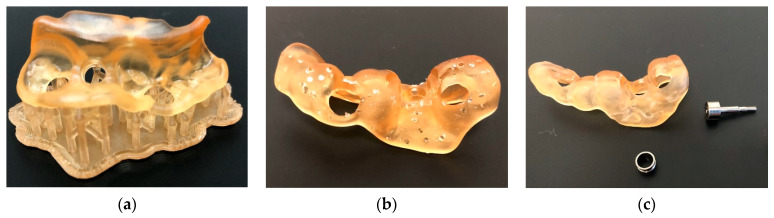
Surgical guide before (**a**) and immediately after removal of the printing support structures (**b**). Additional polishing was required (**c**). The resin used was dental SG (Formlabs, Somerville, MA, USA) printed on Form 3B+ (Formlabs, Somerville, MA, USA).

The more limited the contact that support structures have with the object to be printed, the easier they are to remove (Figure 13). The term “breakaway supports” refers to removal via simple finger pressure. Some resins or printing technologies require thicker supports that must be removed postprinting by milling. In some cases, such as dental models, printing can be performed flat on the build plate without support structures, which saves time and resin [52].

#### 3.4.5. Polishing and Finishing

Polishing and finishing are the steps that still require the most progress to make 3D printing accessible to everyone. This final step often has a significant impact on obtaining the desired properties of the printed element and its long-term aging when used intraorally [79]. Currently, manual steps are systematically involved. Some printing materials are very sensitive to temperature increases during mechanical polishing. It is important to follow the rotation speeds recommended by the manufacturers of polishers and apply low pressure to the instrument. This factor is even more critical for semirigid materials, which are very popular for creating occlusal splints. Indeed, these semirigid materials seem more challenging to polish than rigid materials [80]. However, applying a good polishing protocol allows very good surface states to be achieved. The ability to polish is currently a very important area of research and development for manufacturers to bring 3D printing to a wider audience. A finishing glaze using a photopolymerizable resin (Optiglaze Color, GC Corporation, Tokyo, Japan) can quickly achieve a highly polished surface and improve the aesthetic integration of elements intended for intraoral use.

## 4. Legislation and Recommendations

As 3D printing is relatively recent in the medical field, the related legislation is still in its early stages. There are also significant legislative differences between countries. In some countries, such as Australia, the entire production chain certifies the final quality of the medical device produced, considering each link (biocompatible resin, printer, and postprocessing elements) to be interdependent and capable of impacting the quality of the final product [81]. This scheme is known as the medical device production system (MDPS) concept. Thus, the combination of a specific biocompatible resin with a specific printer and postprocessing chain certifies the quality of the CMD produced by the dentist. This factor often translates into the use of a single-brand printing chain associated with resins certified as compatible with that chain.

In the U.S.A., the Food and Drug Administration (FDA) evaluates a material within the context of a medical product and its intended use and does not clear or approve materials alone for general medical use [82]. No difference is made between the different types of additive manufacturing. Nevertheless, it seems that the entire production chain also certifies the final quality of the medical device produced.

In Europe, obtaining clear positions from relevant authorities on the interpretation of current legislation was challenging, despite several attempts. However, it seems that using a complete printing chain is not a regulatory requirement for certifying the quality and biocompatibility of the CMD produced. Instead, this certification is dependent on the resin used. According to the European Medicines Agency (EMA, Amsterdam, The Netherlands), practitioners must comply with European regulations regarding medical devices [83].

Nevertheless, the following points are common to many countries.

Practitioners wishing to print intraoral prosthetic elements must declare themselves as manufacturers of prostheses to the appropriate local regulatory authorities, just as done by practitioners using subtractive CAD/CAM. Model printing is not affected, but almost all other indications fall under this regulatory obligation.

The routine practice (excluding aesthetics) of the dentist is subject to an obligation of means. The production of custom medical devices (CMDs) using a 3D printer or milling machine shifts the practitioner towards an outcome obligation regarding the production process. This factor does not mean that the therapeutic act itself becomes an outcome obligation but rather that the dentist–manufacturer commits to the quality of the CMDs that are fabricated. In the case of failure due to incorrect procedures, the practitioner can be held responsible.

A practitioner is also required to establish a quality control and traceability system for the prosthetic elements produced. For each CMD produced, the dentist must create a declaration form containing their identity, the technical characteristics of the materials used, and the standards complied with during fabrication. This form must be given to the patient and kept by the practitioner for a fixed number of years, depending on the country. To facilitate the implementation of these time-consuming but essential standards in daily practice, various tools are available, such as the online software CFAO3D, which was developed by a dentist.

Given the aforementioned legislative ambiguity, ethical considerations, and obligation of results for the produced CMDs, a cautious approach is recommended. Therefore, printing CMDs on uncertified printing chains via unapproved printing profiles should be avoided. These uncertified options typically refer to the low-cost LCD printer chains mentioned earlier. A thorough understanding of various postprocessing principles and the application of maximum precautionary measures align with the inherent outcome obligation in CMD fabrication.

## 5. Conclusions

Three-dimensional printing continues to evolve towards the ultimate goal of implementing this technology in daily dental practice. All the technologies available on the market allow for the production of a wide range of medical devices. The choice of technology and brand depends on factors such as the desired printing speed, ease of use, existing digital workflow in the office, and selection of a specific resin.

While the legislation created for the 3D printing of CMDs is not very restrictive, adhering to this legislation and applying the precautionary principles suggested herein could ensure the quality of the produced CMDs. This factor is especially true considering that our knowledge regarding the biocompatibility of the utilized resins remains limited.

## Data Availability

The data that support the findings of this study are available from the corresponding author, [E.C.], upon reasonable request.
